# Small-area deprivation index does not improve the capability of multisource comorbidity score in mortality prediction

**DOI:** 10.3389/fpubh.2023.1128377

**Published:** 2023-05-16

**Authors:** Federico Rea, Mauro Ferrante, Salvatore Scondotto, Giovanni Corrao

**Affiliations:** ^1^National Centre for Healthcare Research and Pharmacoepidemiology, University of Milano-Bicocca, Milan, Italy; ^2^Laboratory of Healthcare Research and Pharmacoepidemiology, Unit of Biostatistics, Epidemiology and Public Health, Department of Statistics and Quantitative Methods, University of Milano-Bicocca, Milan, Italy; ^3^Department of Culture and Society, University of Palermo, Palermo, Italy; ^4^Epidemiologic Observatory, Sicily Regional Health Service, Palermo, Italy; ^5^Directorate General for Health, Lombardy Region, Milan, Italy

**Keywords:** population-based study, comorbidity, prognostic score, healthcare, mortality, socioeconomic position

## Abstract

**Background:**

The stratification of the general population according to health needs allows to provide better-tailored services. A simple score called Multisource Comorbidity Score (MCS) has been developed and validated for predicting several outcomes. The aim of this study was to evaluate whether the ability of MCS in predicting 1-year mortality improves by incorporating socioeconomic data (as measured by a deprivation index).

**Methods:**

Beneficiaries of the Italian National Health Service who in the index year (2018) were aged 50–85 years and were resident in the Sicily region for at least 2 years were identified. For each individual, the MCS was calculated according to his/her clinical profile, and the deprivation index of the census unit level of the individual’s residence was collected. Frailty models were fitted to assess the relationship between the indexes (MCS and deprivation index) and 1-year mortality. Akaike information criterion and Bayesian information criterion statistics were used to compare the goodness of fit of the model that included only MCS and the model that also contained the deprivation index. The models were further compared by means of the area under the receiver operating characteristic curve (AUC).

**Results:**

The final cohort included 1,062,221 individuals, with a mortality rate of 15.6 deaths per 1,000 person-years. Both MCS and deprivation index were positively associated with mortality.

The goodness of fit statistics of the two models were very similar. For MCS only and MCS plus deprivation index models, Akaike information criterion were 17,013 and 17,038, respectively, whereas Bayesian information criterion were 16,997 and 17,000, respectively. The AUC values were 0.78 for both models.

**Conclusion:**

The present study shows that socioeconomic features as measured by the deprivation index did not improve the capability of MCS in predicting 1-year risk of death. Future studies are needed to investigate other sources of data to enhance the risk stratification of populations.

## Introduction

The increasing life expectancy is doubtless one of the highest attainments of the 21st century. On the other hand, aging population has led to a rising number of people affected by comorbidities and consequently a huge demand for healthcare services (1). With the aim of providing better-tailored services and managing expenditure for health assistance, healthcare systems, especially those with universal health coverage, are investigating ways to improve risk stratification of citizens covered by the system (2). The rationale is that by profiling beneficiaries of the health system, namely, by classifying individuals according to their health needs, policymakers can better allocate resources and reduce avoidable events. In addition, identifying the number of individuals who need tailored services should improve appropriate and timely healthcare for frail citizens and improve their prognosis.

A way to identify frail individuals who require tailored services is through the concept of multimorbidity (3). Although the measurement of multimorbidity is highly variable and there is no universally agreed definition (4), numerous scores have been developed (5). Models are usually based on the individual count of chronic conditions from which every beneficiary of the health system suffers, in identifying those independently associated with the risk of certain adverse outcomes (e.g., mortality, healthcare use, and costs), and in weighing them according to the strength of the association between each of them with the outcomes of interest. However, as socioeconomic position is known to be associated with the development of a range of physical illnesses (6) and mortality (7), chronic conditions should be considered together with other potential predictors of adverse outcomes. Nevertheless, risk stratification tools usually do not account for socioeconomic conditions (5), which raises the question of whether the prediction of an individual’s outcomes can be improved by jointly accounting for socioeconomic conditions and multimorbidity status.

With this premise, a real-world investigation has been carried out to answer the following two questions: (i) does a model that considers the relationship between multimorbidity and clinical outcomes improve by incorporating socioeconomic position? and (ii) does social and material deprivation modify the relationship between multimorbidity and clinical outcomes?

## Materials and methods

### Setting

This study included data from healthcare utilization databases of Sicily, a region of Italy that accounts for approximately 8% of the country’s population (almost 5 million individuals). In Italy, the whole population is covered by the National Health Service (NHS), and an automated system of databases exists in each region aimed at collecting a variety of information, including demographic and administrative data of residents, admissions in public and private hospitals (primary diagnosis, coexisting conditions and procedures), and outpatient drug prescriptions. As for many other international settings, socioeconomic features are not collected in these databases.

### Cohort selection

Individuals who in the index year (2018) were aged 50–85 years and were resident in Sicily for at least 2 years (i.e., who were recorded as beneficiaries of the Regional Health Service before 2016) were identified. Among these, those for whom it was possible to link census data (see below) formed the study population. The individuals included in the study cohort accumulated person-years of follow-up from January 1st, 2018 until the earliest date between death (outcome of interest), emigration or January 1st, 2019.

### Multisource comorbidity score

The Multisource comorbidity score (MCS) was developed and validated in Italy for predicting several outcomes (mortality, hospital admissions and healthcare costs) (8). For each individual in the study cohort, the 34 diseases/conditions contributing to MCS were traced from footprints left by NHS beneficiaries through diagnostic codes in hospital records and drugs dispensed in community pharmacies. A weight was assigned for each disease/condition according to the strength of the corresponding condition in predicting 1-year mortality. MCS was then calculated as the sum of the weights of the diseases/conditions identified for each individual. Further information can be found in the original paper (8). MCS showed better discriminatory power than other commonly used prognostic scores [i.e., Charlson comorbidity index, Elixhauser index and Chronic Disease Score (9–11)].

The individual record reporting the MCS value of each cohort member, jointly with his/her sex, year of birth, and coordinates of residence place, were obtained for the current application.

### Deprivation index

The Deprivation Index (DI) was developed from the Italian census data^1^ by considering five socioeconomic traits such as low level of education, unemployment, non-home ownership, single-parent family, and overcrowding (12, 13). A positive association with mortality was reported for the DI. Details on methods to calculate the DI, comprehensive of recent updating introduced for overcoming certain limitations of the original version, are reported in the Appendix.

DI calculated according to the last available year of the Italian census (2011) was considered for the current application. With the aim of accounting for excessive heterogeneity of the index, it was categorized by assigning increasing values of 1, 2, 3, and 4. With the aim of overcoming the known limitations in the use of quantile categories (14), we used the method of Jenks natural breaks optimization, which identifies break points that maximize the differences between classes (15).

Data on DI was collected at the census unit level. Census units are the smallest administrative areas that divide the Italian territory and cover an average of 150 inhabitants each.

### Data analysis

With the aim of investigating the association between MCS profile and DI, the age above which half of the beneficiaries suffered from at least one comorbidity (MCS median age) and the across-age average percentage of people with at least one comorbidity (percentage of comorbid population) were calculated according to DI categories (16).

As MCS was available at an individual level, whereas DI data was available at the census unit level, the population under analysis presented a clear hierarchical structure with individuals (level 1) nested within the census unit (level 2) (17). In the framework of survival analysis, models including random effects are denoted as frailty models (18). For assessing whether frailty models better fit than conventional Cox proportional hazards model with fixed effects, we used the likelihood ratio test (19). Because the null hypothesis of equivalence between models was rejected (with a *p*-value of 0.010), the hierarchical structure of data was not ignored in our application. Therefore, random intercept models were fitted assuming that the distribution of the frailty term followed a gamma distribution. With this approach, all individuals in the same census unit had an increase/decrease in the hazard of death.

With the aim of assessing whether DI may improve the prediction of mortality based on the MCS-based model, both Akaike and Bayesian information criterion statistics (AIC and BIC respectively) were used to compare the goodness of fit of models including MCS only and MCS jointly with DI terms. AIC and BIC, based on log-likelihood and the number of parameters, can be used to compare the goodness of fit by taking into account the model’s complexity. The best model is the one with the lowest values of AIC and BIC. In addition, the discriminatory power of the two models was compared through the corresponding receiver operating characteristic (ROC) curves and the area under the ROC curve (AUC) (20).

With the aim of evaluating whether DI modified the relationship between MCS and mortality, three analyses were performed. First, frailty models were fitted to estimate the hazard ratios and 95% confidence intervals of death associated with MCS by stratifying for the DI categories. Heterogeneity between DI categories (i.e., the DI effect modifying capability) was tested by Cochran’s Q test and measured with the I^2^ statistic (21). Second, a frailty model was fitted by including MCS, DI, and the interaction term between MCS and DI. Third, the ROC curves and corresponding AUCs of MCS were calculated within each category of the DI.

Statistical analyses were performed within the R software environment.

## Results

### Study cohort and scores

Among the 1,617,002 eligible cohort members, the availability of census data was obtained for 1,062,221 of them (65.7%), the latter being included in the study cohort. There was no evidence that included individuals were selected with respect to eligible ones according to selected features (Supplementary Table S1) and mortality rates (being the latter values of 15.6 and 15.8 per 1,000 person-years among citizens included and excluded from the study cohort, respectively).

**Figure 1:**
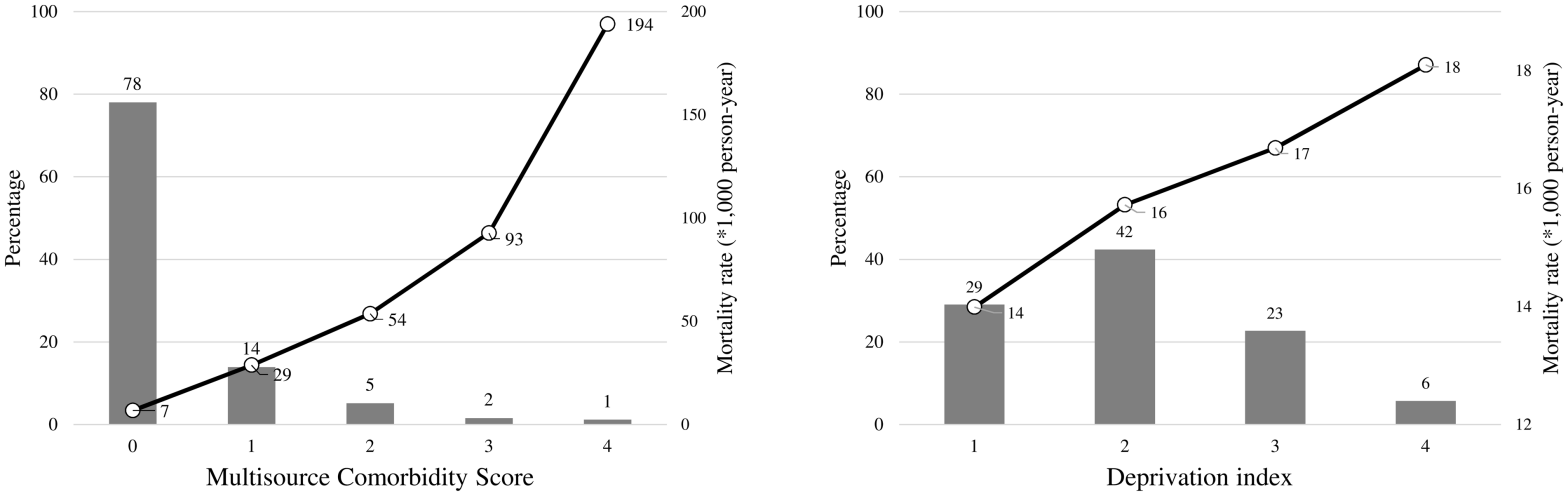
Distributions of the multisource comorbidity score and the deprivation index among National Health Service beneficiaries, and 1-year mortality rates observed in each category of the scores. Multisource Comorbidity Score was categorized according to the classes defined in the original manuscript (8): 0 (score 0–4), 1 (5–9), 2 (10–14), 3 (15–19), and 4 (≥20).

Overall, 78 and 1% of citizens had the lowest (0) and the highest (4) MCS value, respectively, while 6% of cohort members belonged to the worst DI category (Figure 1). The mortality risk was positively associated with both indexes. Indeed, the mortality rate ranged between 7 and 194 per 1,000 person-years along categories of MCS, and from 14 to 18 per 1,000 person-years for DI.

**Figure 2:**
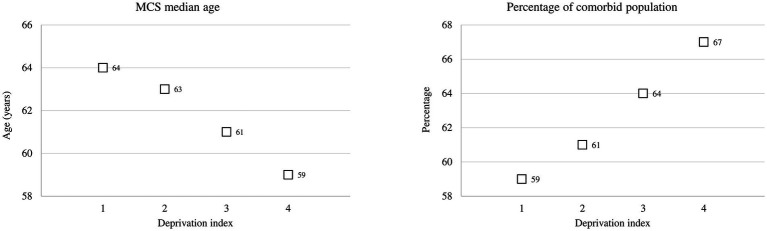
Multisource comorbidity score (MCS) median age and the percentage of the comorbid population among categories of the deprivation index.

There was a positive association between DI and MCS. As shown in Figure 2, MCS median age decreases as raising DI, whereas the opposite occurred for the percentage of comorbid population.

### The added value of the deprivation index

**Figure 3:**
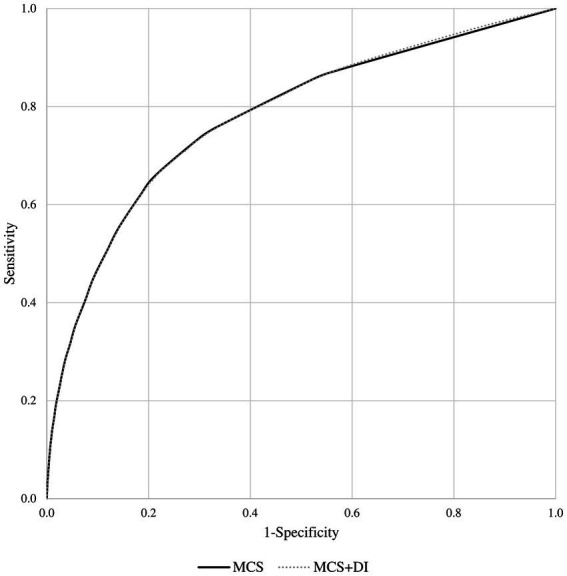
Receiver operating characteristic (ROC) curves comparing the discriminant power of the multisource comorbidity score (MCS) only and the MCS plus deprivation index (DI) models in predicting 1-year survival among National Health Service beneficiaries.

The model including only MCS showed very similar goodness-of-fit statistics as the model including both MCS and DI terms (being the corresponding AIC and BIC statistics 17,013 and 17,038, and 16,997 and 17,000). The estimates of the risk of death according to the two models are shown in Tables 1, 2. Similarly, the discriminant power of models including MCS only and both MCS and DI terms was practically the same, having AUC values of 0.78 for both models (Figure 3). The 95% confidence intervals were not reported because, owing to the very large sample size, they coincided with the AUC values.

**Table 1:** Hazard Ratio (HR) and 95% confidence interval (CI), for all-cause death associated with Multisource Comorbidity Score.

	HR (95% CI)
Multisource Comorbidity Score	1.116 (1.114–1.117)

Akaike information criterion: 17,013; Bayesian information criterion: 16,997.

**Table 2:** Hazard Ratio (HR) and 95% confidence interval (CI), for all-cause death associated with Multisource Comorbidity Score and Deprivation index.

	HR (95% CI)
Multisource comorbidity score	1.116 (1.114–1.117)
Deprivation index	
1	1.000 (Ref.)
2	1.081 (1.038–1.125)
3	1.119 (1.069–1.171)
4	1.154 (1.076–1.239)

Akaike information criterion: 17,038; Bayesian information criterion: 17,000.

### MCS and mortality among categories of the deprivation index

**Figure 4:**
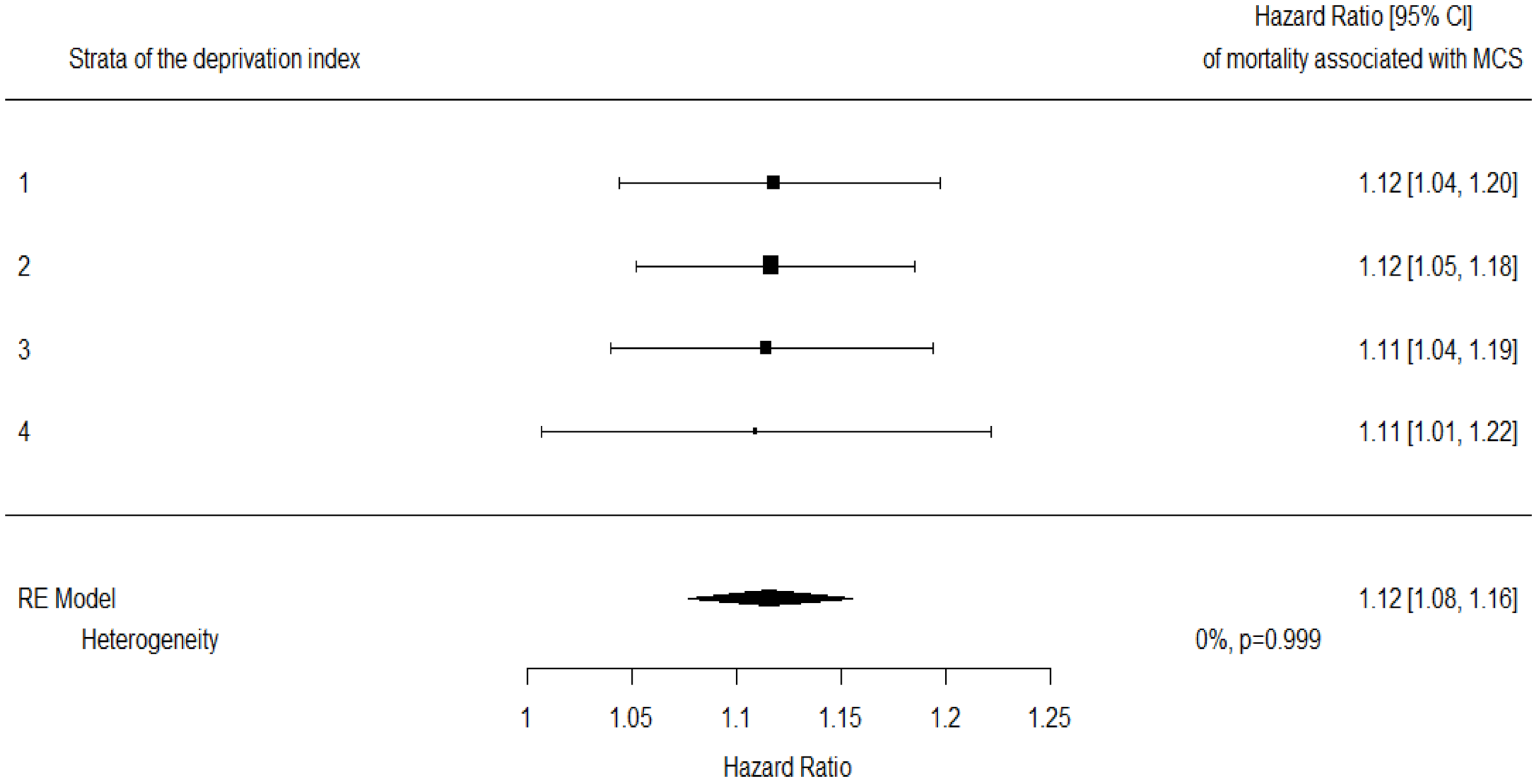
Hazard ratios for mortality associated with multisource comorbidity score (MCS) among categories of the deprivation index and a summarized estimate. Hazard ratios measuring the increased mortality risk associated with each additional point of MCS.

**Figure 5:**
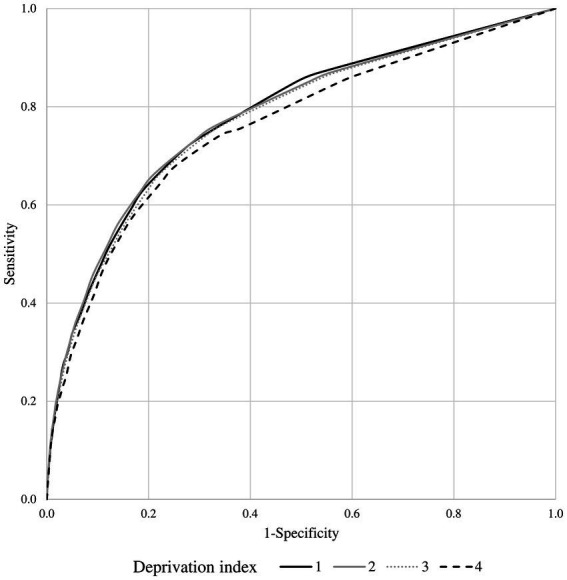
Receiver operating characteristic (ROC) curves comparing the discriminant power of multisource comorbidity score (MCS) in predicting 1-year survival among the categories of the deprivation index.

The association estimates between MCS and mortality according to the DI are shown in Figure 4. According to the summarized estimates, each additional point of MCS was associated with a 12% increased mortality risk (95% CI 8–16%). There was no evidence that the association between MCS and mortality differed between strata of the DI (I^2^ = 0%, *p*-value = 0.999). This was confirmed by including the interaction term between MCS and DI in a frailty model (*p*-value = 0.130). AUC values associated with the discriminant power of MCS were 0.78, 0.78, 0.77, and 0.76 from the first to the last DI category (Figure 5).

## Discussion

The present study shows that socioeconomic features (as measured by the DI) neither improved the capability of MCS in predicting 1-year risk of death nor modified the relationship between multimorbidity and clinical outcomes.

Several aspects of the present study deserve to be mentioned to better understand the public health implications. First, consistently with published evidence in this field (22–25), it results that the higher the small-area social deprivation, the greater healthcare needs in that area. Second, our findings are also consistent with previous observations that mortality is higher among individuals who live in areas on social deprivation (26, 27). Third, as the predictive capability of the model including only MCS did not improve by adding the average social deprivation, comorbidity almost completely absorbed the effect of deprivation on mortality. Because comorbidity may be believed as a mediator of the relationship between socioeconomic position and mortality (28), the finding is not surprising. Finally, the predictive capability of the MCS did not change between areas with different categories of deprivation. This is consistent with previous reports from Italy showing that comorbidity scores predicted mortality, and other adverse outcomes, homogeneously among the Italian regions (despite the notable socio-economic heterogeneity among the regions) (8) and among individual income (29). Taken together, these findings suggest that MCS can capture the health needs of a population covered by universal health coverage irrespective of the socioeconomic profile.

The study’s strengths include the very large population and the use of a validated index of comorbidity (8, 16) and deprivation (12, 13). Nevertheless, some limitations should be declared. First, as socioeconomic data at an individual level was not available, data collected from the national census at the census tract level were used. Although census tract levels include few individuals (on average 150 inhabitants), social and material deprivation could vary among individuals in the same administrative area. In addition, although the contribution of the contextual deprivation was confirmed by others (30), future research should investigate the specific role of individual deprivation on healthcare needs. Second, it was possible to link the census data information for 66% of the target population. Although there was no evidence that included cohort members differed with respect to eligible ones, selection bias cannot be wholly excluded, even its effect on our findings should be likely modest. Third, because the last Italian census was made in 2011, available deprivation data lagged seven-eight years behind multimorbidity information (available for the years 2018–2019). However, because socioeconomic features are not expected to vary quickly, their asynchrony with morbidity data likely generates not overly relevant uncertainty. Finally, the generalizability of our findings to other Italian regions requires caution. Because between-region heterogeneity of socioeconomic profile is expected to be high, the added value of the DI should be better investigated including other Italian regions with different socioeconomic patterns.

In conclusion, MCS represents a useful tool that policymakers can use to stratify NHS beneficiaries according to the risk of several outcomes (mortality, hospital admissions and healthcare costs). Socioeconomic data, as measured by the DI, did not improve the risk prediction of MCS, nor modify the effect of MCS on mortality. Future studies are needed to investigate other sources of data to enhance the population risk stratification.

## Disclosures

GC received research support from the European Community (EC), the Italian Medicines Agency (AIFA), Italian Ministry of Health, and the Italian Ministry of Education, University and Research (MIUR). He took part to a variety of projects that were funded by pharmaceutical companies (i.e., Novartis, GSK, Roche, AMGEN, BMS and Servier). He also received honoraria as member of Advisory. The other authors report no disclosures.

## Data availability statement

The data analyzed in this study is subject to the following licenses/restrictions: The data that support the findings of this study are available from Sicily Region, but restrictions apply to the availability of these data, which were used under license for the current study, and so are not publicly available. Data are however available from the Sicily Region upon reasonable request. Requests to access these datasets should be directed to walter.pollina.ext@regione.sicilia.it.

## Author contributions

FR and GC designed the study. SS contributed to the critical interpretation and discussion of the results. FR and MF performed data analysis and wrote the manuscript. SS and GC reviewed the manuscript. All authors contributed to the article and approved the submitted version.

## CHRP-Sicily Region working group

National Centre for Healthcare Research and Pharmacoepidemiology (CHRP) – Sicily Region working group: Sicily Region: Salvatore Scondotto, Sebastiano Pollina Addario, Giovanna Fantaci, Alessandra Allotta, Giovanni De Luca, Elisa Tavormina, Pasquale Cananzi, Achille Cernigliaro, Francesco La Placa. University of Milano-Bicocca: Giovanni Corrao, Federico Rea, Anna Cantarutti, Matteo Monzio Compagnoni. University of Palermo: Mauro Ferrante, Domenica Matranga, Laura Maniscalco, Andrea Mattaliano. University of Catania: Antonella Agodi, Martina Barchitta. University of Messina: Ylenia Ingrasciotta, Valentina Isgrò. Polytechnic University of Marche: Flavia Carle, Edlira Skrami, Marica Iommi. Research and Healh Foundation (Fondazione ReS – Ricerca e Salute): Nello Martini, Antonella Pedrini.

## Funding

This study was funded by grants from the Sicily Region [“Messa a punto di un modello di valutazione della qualità dei percorsi di gestione integrata per alcune condizioni di cronicità” project, grant number H45J19001550002], Italian Ministry of Health [“Modelli per il monitoraggio e la valutazione delle cure integrate (CI) nell’ambito del Nuovo Sistema di Garanzia dell’assistenza sanitaria” project, grant number J59H06000160001] and Italian Ministry of the Education, University and Research [“Fondo d’Ateneo per la Ricerca,” year 2020, grant number 2020-ATE-0541].

## Conflict of interest

The authors declare that the research was conducted in the absence of any commercial or financial relationships that could be construed as a potential conflict of interest.

## Publisher’s note

All claims expressed in this article are solely those of the authors and do not necessarily represent those of their affiliated organizations, or those of the publisher, the editors and the reviewers. Any product that may be evaluated in this article, or claim that may be made by its manufacturer, is not guaranteed or endorsed by the publisher.

## Footnotes

^1^ https://www.istat.it/en/
