# Supplementary material for: Small-area deprivation index does not improve the capability of multisource comorbidity score in mortality prediction
Source: Front Public Health. 2023 May 16;11:1128377. doi: 10.3389/fpubh.2023.1128377 (PMC10228715; doi:10.3389/fpubh.2023.1128377)
Supplement: Supplementary file 1 [file Table_1.DOCX]

**Small-area deprivation index does not improve the capability of multisource comorbidity score in mortality prediction**

Federico Rea^1,2^, Mauro Ferrante^3^, Salvatore Scondotto^4^, Giovanni Corrao^1,2,5^; on behalf of the CHRP-Sicily Region working group

^1^ National Centre for Healthcare Research and Pharmacoepidemiology, University of Milano-Bicocca, Milan, Italy

^2^ Laboratory of Healthcare Research and Pharmacoepidemiology, Unit of Biostatistics, Epidemiology and Public Health, Department of Statistics and Quantitative Methods, University of Milano-Bicocca, Milan, Italy

^3^ Department of Culture and Society, University of Palermo, Palermo, Italy

^4^ Epidemiologic Observatory, Sicily Regional Health Service, Palermo, Italy

^5^ Directorate General for Health, Lombardy Region, Milan, Italy

**Supplementary Material**

**Appendix**

The Italian deprivation index was developed based on the following five traits:

- low level of education: proportion of individuals with less than 5 years of education among those aged 6 years or older,
- unemployment: proportion of individuals in the labour force who do not currently have a job,
- non-home ownership: proportion of dwellings lived in by tenants,
- single-parent family: proportion of families with one parent,
- overcrowding: population density defined as the ratio between the number of people and the area occupied by dwellings.

In each census section level (i.e., administrative area which includes on average 150 inhabitants), these data are recorded. The deprivation index is calculated as the sum of the above-mentioned variables after their standardization (i.e., each variable was subtracted from the mean and divided by the standard deviation) [1].

The index has, however, two limitations. First, the indicator of “low education” includes in the denominator children aged less than 10 years (who cannot reach 5 years of education) and elderly people (who usually have a low education). Therefore, this indicator is affected by the number of children and elderly living in that census section level. Second, the “single-parent family” indicator does not take into account the age of the child and thus the variable does not discriminate between minor and adult sons. To overcome these limitations, the deprivation index was recently updated [2] as follows: (i) the indicator regarding the “low education” is now calculated among individuals aged 15-60 years, and (ii) the “single-parent family” indicator is now based on only families with minor children.

**References**

[1] Caranci N, Biggeri A, Grisotto L, Pacelli B, Spadea T, Costa G. The Italian deprivation index at census block level: definition, description and association with general mortality. Epidemiol Prev. 2010;34:167-76

[2] Rosano A, Pacelli B, Zengarini N, Costa G, Cislaghi C, Caranci N. Update and review of the 2011 Italian deprivation index calculated at the census section level. Epidemiol Prev. 2020;44:162-170. Italian

**Supplementary Table S1**. Characteristics of individuals included and excluded from the study cohort

|  | **Included**  **(N=1,062,221)** | **Excluded**  **(N=554,781)** | **Standardized**  **difference** |
| --- | --- | --- | --- |
| Men | 489,669 (46.1%) | 262,223 (47.3%) | 0.023 |
| Age: mean (standard deviation) | 64.6 (9.8) | 64.5 (9.9) | 0.010 |
| Multisource Comorbidity Score |  |  |  |
| 0 | 829,359 (78.1%) | 427,953 (77.1%) | 0.023 |
| 1 | 148,086 (13.9%) | 82,087 (14.8%) | 0.024 |
| 2 | 55,188 (5.2%) | 29,446 (5.3%) | 0.005 |
| 3 | 16,880 (1.6%) | 8,876 (1.6%) | 0.001 |
| 4 | 12,708 (1.2%) | 6,419 (1.2%) | 0.004 |

MCS was categorized according to the following categories: 0 (score 0-4), 1 (5-9), 2 (10-14), 3 (15-19), and 4 (≥20).
